# Cutaneous larva migrans: a case report

**DOI:** 10.1186/1757-1626-2-112

**Published:** 2009-01-31

**Authors:** Sergio Vano-Galvan, Manuel Gil-Mosquera, Mayte Truchuelo, Pedro Jaén

**Affiliations:** 1 Department of Dermatology, Ramon y Cajal Hospital, University of Alcala, Madrid, Spain Carretera Colmenar km 9.100 28034 Madrid, Spain; 2 Family physician, Ramon y Cajal Hospital, University of Alcala, Madrid, Spain

## Abstract

**Background:**

Cutaneous larva migrans may be diagnosed by the typical clinical presentation, consisting on a pruritic serpiginous lesion that advances in a patient with a history of sunbathing, walking barefoot on the beach, or similar activity in a tropical location.

**Case presentation:**

We describe the case of a Mediterranean 32-year-old man, recently returned from a trip to a Brazilian beach, which presented with a 2-week history of pruritic cutaneous lesions that had not resolved after treatment with oral antihistamines and topical fluocinolone ointment. Physical examination showed a serpiginous, erythematous and slightly elevated lesion of 2-mm wide and 7-cm long located on the posterior aspect of his left knee. Patient affirmed that the lesion advanced progressively. Laboratory analyses only revealed an elevated absolute eosinophil count. Albendazole 400 mg/d 3 days was administered to the patient with complete resolution of symptoms within 1 week.

**Conclusion:**

Cutaneous larva migrans is common among travelers returning from tropical countries. We review epidemiology, clinical, diagnosis and therapeutic options of cutaneous larva migrans.

## Case presentation

A Mediterranean 32-year-old man was referred to our department for a 2-week history of pruritic cutaneous lesions that had not resolved after treatment with oral antihistamines and topical fluocinolone ointment. He had recently returned from a trip to a Brazilian beach. He had no other symptoms and was otherwise well. His medical history was irrelevant.

**Figure 1:**
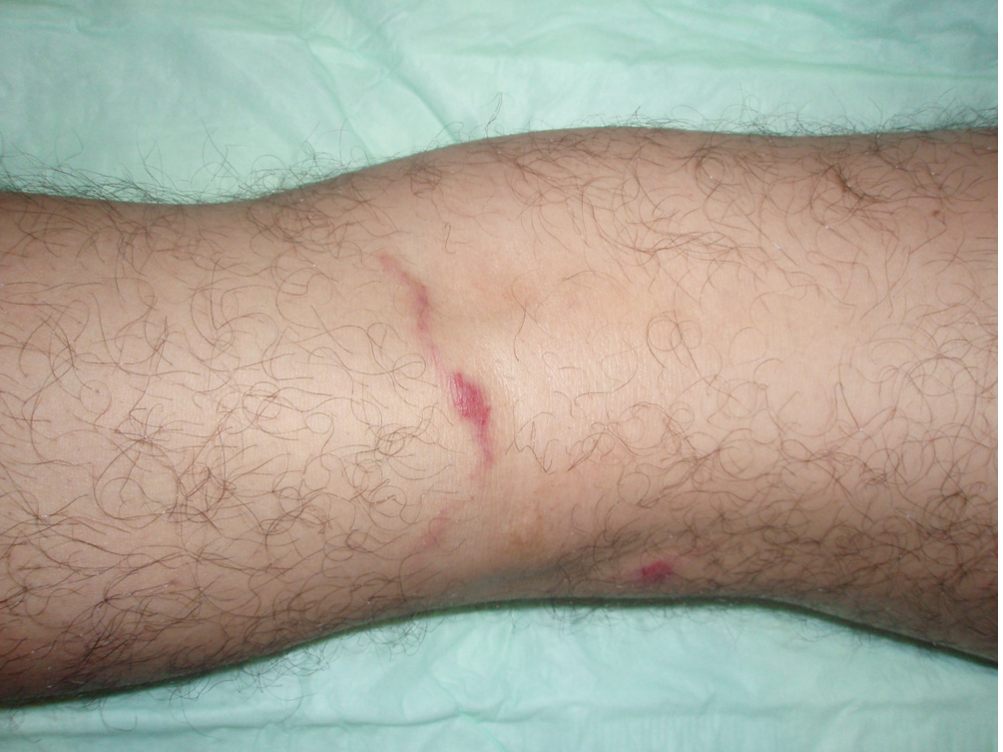
**Dermatological examination showed an erythematous serpiginous intraepidermal tunnel on his left leg, caused by accidental percutaneous penetration of the larva**.

Physical examination showed a serpiginous, erythematous and slightly elevated lesion of 2-mm wide and 7-cm long located on the posterior aspect of his left knee (Figure 1). Patient affirmed that the lesion advanced progressively. The remainder of his physical examination was within normal limits. Laboratory analyses only revealed an elevated absolute eosinophil count (1324 × 10^9^/L).

With the strong clinical suspect of cutaneous larva migrans, albendazole 400 mg/d 3 days was administered to the patient with complete resolution of symptoms within 1 week.

## Discussion

Cutaneous larva migrans – also known as creeping eruption or sandworm disease – is common among travelers returning from tropical countries. It is caused by larvae of animal hookworms. Ancylostoma braziliense is the most common offender [1].

The disease is endemic in resource-poor communities in the developing world, particularly in Brazil, India, and the West Indies. It occurs sporadically or in the form of small epidemics in high-income countries and is reported in tourists who have visited the tropics [2].

Most often, people pick up the infection by walking through contaminated areas barefoot or with open-type shoes, or by sitting in tainted soil or sand [1].

The main affected areas are the dorsum and sole of the feet (uni and bilateral), buttocks, pelvic waist, legs and shoulders. More than one lesion is compatible with more than one entry point. The main signs and symptoms are linear and/or serpiginous lesions (which progress from 2–3 mm to 2–3 cm per day) and the pruritus. This is intensified after some days by the inflammatory reaction of the host and may even interfere with sleep. Pain may occur in papulovesicular lesions. Systemic signs include peripheral eosinophilia (Loeffler syndrome), migratory pulmonary infiltrates, and increased immunoglobulin E levels, but are rarely seen.

The diagnosis of hookworm-related cutaneous larva migrans is easily made clinically on the basis of typical clinical presentation which is a pruritic serpiginous lesion that advances in a patient with a history of sunbathing, walking barefoot on the beach, or similar activity in a tropical location. Creeping eruption as a clinical sign is diagnostic; a biopsy is not useful.

Even though the condition is self-limited, the intense pruritus and risk for infection mandate treatment. Different therapeutic approaches are effective: a single dose of ivermectin (200 μg per kg bodyweight) kills the migrating larvae effectively and relieves itching quickly. Oral albendazole (400 mg daily), given for 5–7 days, shows excellent cure rates and the drug is well-tolerated by patients [3]. Thiabendazole (50 mg per kg bodyweight for 2–4 days) was widely used after the first report of its efficacy in 1963. However, given orally the substance is poorly tolerated, and frequently causes dizziness, nausea, vomiting, and intestinal cramps [2]. Topical thiabendazole 10% cream, although less effective, is a good alternative for young children to avoid the potential side effects of systemic medications.

The prognosis is excellent. This is a self-limiting disease. Humans are accidental, dead-end hosts, with the larva dying and the lesions resolving within 4–8 weeks, as long as 1 year in rare cases.

In prevention, when visiting tropical countries, especially beaches and sandy, moist areas, it is best to wear shoes that completely cover the feet. Also, one should avoid sitting or lying on bare sand, even if on a towel. Deworming of pets is recommended [1].

## Consent

Written informed consent was obtained from the patient for publication of this case report and accompanying images. A copy of the written consent is available for review by the Editor-in-Chief of this journal.

## Competing interests

The authors declare that they have no competing interests.

## Authors' contributions

SVG wrote the initial draft of and helped revise the manuscript. MGM and MT obtained consent from the patients and helped revise the manuscript. PJ assisted with manuscript revision. All authors read and approved the final manuscript.
